# In silico study of the sensing properties of C18, B9N9, and Al9N9 nanorings for diabetes monitoring via indole detection in exhaled breath

**DOI:** 10.1038/s41598-025-33074-8

**Published:** 2025-12-24

**Authors:** Abdulwahab Alamri, Ahmed Alafnan

**Affiliations:** Department of Pharmacology and Toxicology, College of Pharmacy, University of Ha’il, 55476 Hail, Saudi Arabia

**Keywords:** Indole sensing, Volatile organic compounds (VOCs), Nanorings (C_18_, B_9_N_9_, Al_9_N_9_), Density functional theory (DFT), Non-invasive diabetes diagnosis, Chemistry, Nanoscience and technology

## Abstract

Detecting indole in exhaled breath has immense promise for non-invasive diabetes monitoring. Here, we pursued a computational evaluation of the performance of C_18_, B_9_N_9_, and Al_9_N_9_ nanoring structures as sensors for indole. The evaluated processes included Density Functional Theory calculations, Quantum Theory of Atoms in Molecules analysis, Non-Covalent Interaction studies, and UV–Vis spectral analysis, to interpret electronic interactions and adsorption between indole and sensors. The findings suggested that B9N9 showed the most significant interaction with indole, with the highest adsorption energy (− 20.71 kcal/mol) and longest recovery time (1.54 × 10^3^ s), indicating its superior sensitivity. Al_9_N_9_ performed moderately, while C18 had the lowest interaction with indole. Furthermore, B_9_N_9_ showed a significant increase in electrical conductivity (2.81 × 10^9^–2.88 × 10^9^ S/m), making it an attractive candidate for electrochemical sensors. In the UV–Vis spectra, Al_9_N_9_@Indole showed a significant red shift of 87 nm, going from 376 to 463 nm. This change shifts it from the UV range into the visible spectrum, enabling a noticeable color change. Therefore, B_9_N_9_ is well-suited for electrochemical sensing due to its high sensitivity and fast response. Al_9_N_9_ is notable for its visible optical response, which makes it suitable for colorimetric sensing when indole is present.

**Supplementary Information:**

The online version contains supplementary material available at 10.1038/s41598-025-33074-8.

## Introduction

Diabetes mellitus is a common chronic metabolic disease seen throughout the world, and it is defined by hyperglycemia that persistently occurs due to inadequate insulin secretion, insulin resistance, or both. There are millions of individuals with diabetes and related serious complications of the body, particularly cardiovascular disease, neuropathy, nephropathy, and retinopathy. These complications diminish quality of life while also increasing costs to the healthcare system. Current and past global health reports document that the incidence of diabetes is rapidly increasing, demonstrating the need for early detection while managing the disease to lower morbidity and mortality^1,2^. Although tests such as fasting blood sugar, oral glucose tolerance, and HbA1c are valid criteria for diagnosing diabetes, they are usually invasive and expensive. They also usually only reveal the presence of the diabetic disease process when significant metabolic changes have occurred^3,4^. Both of these issues present opportunities for early detection using noninvasive biomarker measurements. In this regard, volatile organic compounds (VOCs) have been increasingly viewed as possible non-invasive biomarkers of dysregulated metabolism. Indole, a metabolite that is produced by gut microbiota from the catabolism of tryptophan, was recently detected in the exhaled breath of diabetic individuals. Fink et al. suggested that levels of indole might be a potential candidate for the non-invasive monitoring of blood glucose levels, providing a new and interesting pathway in the field of breath-based diagnostics of diabetes. This important result suggests that indole might not only serve as a biomarker for host-microbiome interaction, but also represents a new research avenue toward developing novel, accessible, and easy-to-use diagnostic technologies for patients^5^.

Recent advances in nanosensor technology have improved the detection of biomarkers. For example, Wang et al. highlighted the role of graphene nanosensors in the development of biomedical sensors^6^. Li et al. developed a FET-type biosensor for the detection of Alzheimer’s disease biomarkers^7^. Yudsin et al. also highlighted the potential of MOF metal–organic frameworks as a sensing platform for COVID-19 biomarkers^8^. The studies emphasize the increasing application of nanomaterials and biosensing techniques in contemporary diagnostic technologies. For the case of diabetes, such sensor technologies could potentially work in tandem with developing biomarkers, such as indole, and lead to sensitive, selective, and non-invasive platforms for disease detection and monitoring. Studies show that carbon nanomaterials have played a pivotal role in the advancement of sensor and drug delivery technology. Outside of existing systems such as fullerenes, carbon nanotubes, and graphene, recent publications have introduced cyclo[n]carbons, particularly cyclo[18]carbon (C_18_), because of its geometrical and electronic structure. After Gavel et al. synthesized C_18_, the electronic properties that arise from the large π-conjugation made C_18_ an obvious candidate for sensing^9^. Boron nitride (B_9_N_9_) and aluminum nitride (Al_9_N_9_) nanorings, which are isoelectronic with C_18_, have attracted attention due to their stability and electronic properties such as wider band gap and higher reactivity^10^. Since experimental evidence confirms the stability of each of these structures, theoretical studies emphasize the sensing application of C_18_, B_9_N_9_, and Al_9_N_9_^11^. For example, Sajid et al. highlighted the utility of monocyclic C18 and B9N9 as active materials for sensing and removing chemical warfare agents (CWAs) such as formaldehyde (Fd), phosgene (Ph), and thiophosgene (TPh)^12^. Niamat et al. showed that both C_18_ and B_9_N_9_ are suitable for electrochemical sensing and removal of anticancer derivatives (carbazole-based)^13^. Also, Panchal et al. emphasized the sensing application of B_9_N_9_ and Al_9_N_9_ in the adsorption and detection of hazardous gas molecules CO, NO and NH_3_^14^. Based on these studies, it is expected that C_18_, B_9_N_9_ and Al_9_N_9_ nanorings can exhibit acceptable performance in adsorption/sensing applications. Therefore, considering the unique electronic and structural properties of C_18_, B_9_N_9_, and Al_9_N_9_ nanorings and the need for a rapid and non-invasive method for diabetes diagnosis, this study aims to perform a sensing test of these nanorings for indole detection that can be measured in the exhaled breath of diabetic patients. In this study, we test the interaction between indole molecules and the entire nanostructured platform to question the sensitive and selective nature of early diabetes symptom detection.

Due to the speed, multiple studies, and rapid availability of results, computational methods are usually considered as a pre-synthesis step to evaluate/predict molecular interactions. DFT (density functional theory) and QTAIM (quantum theory of atoms in molecules) are useful computational approaches to study such molecular interactions, electronic properties, and adsorption mechanisms at the molecular scale^15,16^. In this study, we present DFT and QTAIM analyses for the design of efficient sensors based on C_18_, B_9_N_9_, and Al_9_N_9_ nanorings for the detection of indole (biomarker of diabetes). We aim to use the results of this computational study to help researchers develop and synthesize inexpensive, efficient, sensitive, and selective biosensors for rapid and non-invasive diagnosis of diabetes.

## Computational method

**Figure 1:**
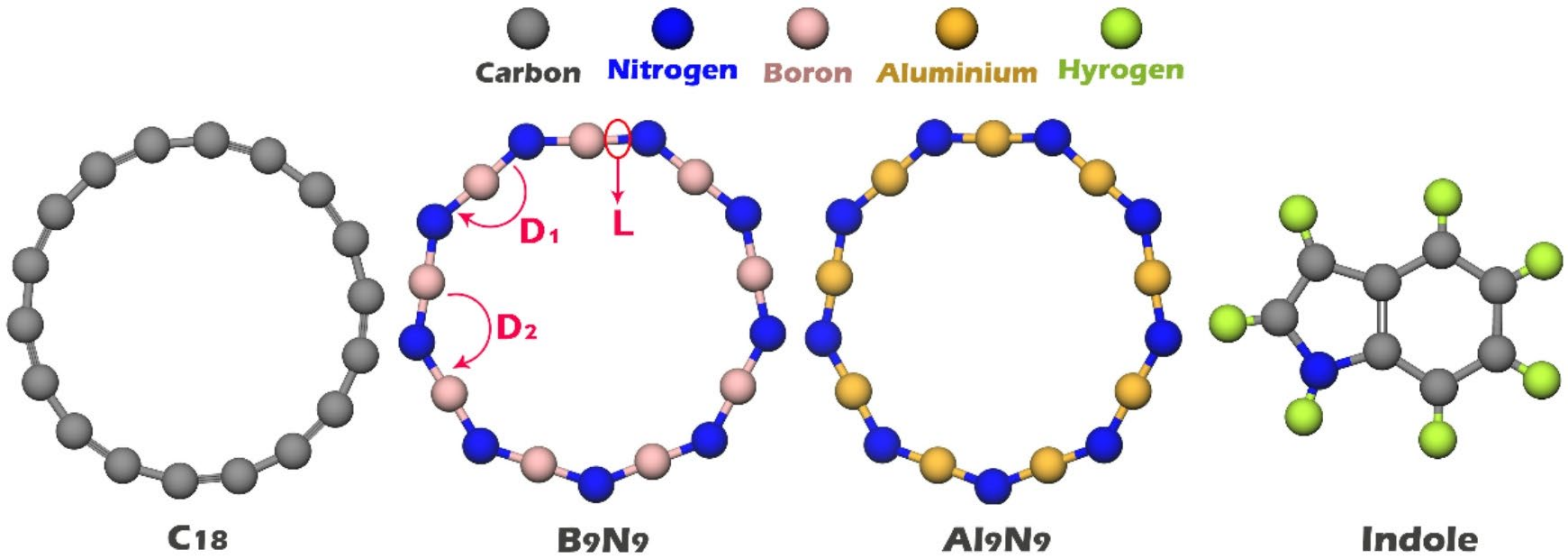
Molecular structure of indole and C_18_, B_9_N_9_, and Al_9_N_9_ nanorings.

Initially, all structures (Indole, C_18_, B_9_N_9_, Al_9_N_9_, C_18_@Indole, B_9_N_9_@Indole, and Al_9_N_9_@Indole) were designed and optimized using GaussView 6.0 and Gaussian 09W software, respectively^17^. Geometric optimization was performed in the aqueous phase (using the Conductor-like Polarizable Continuum Model (CPCM) to account for air humidity) and at the B97D/6-311G(d) computational level (See Fig. 1)^18^. Also, in order to verify the accuracy of the chosen computational approach, the structure of each nanoring was optimized in isolation using the WB97XD/6-311G(d) computational method. The results obtained from this method were compared with the results obtained from the B97D/6-311G(d) computational method (for nanorings in the absence of indole).

The structural features of each optimized geometry (calculated from both WB97XD/6-311G(d) and B97D/6-311G(d) methods), such as the minimum energy diagram (Fig. S1), bond length/angle (Table S1), cohesive energy values (Table S2), and IR spectra (Fig. S2) are listed in the Supplementary Data. The obtained results showed a very good overlap. For this purpose, the B97D functional with the 6-311G(d) basis set was considered for further calculations.

The B97d functional was chosen due to its reliable description of dispersion interactions and non-covalent forces, which are crucial in nanosensor-biomarker interactions^19^. Furthermore, previous studies have shown that B97D is in good agreement with experimental data for other carbon-based structures^20,21^. Frequency calculations were performed at the same theoretical level, and no imaginary frequencies were observed, indicating that the designed structures are stable and not in a transition state. Time-dependent DFT (TD-DFT) calculations and the same computational level were also used to calculate the UV–Vis spectra of each of the studied structures in the presence/absence of indole^22^. The electronic properties of each structure, including energy gap (HLG = E_HOMO_-E_LUMO_), chemical hardness (η = HLG/2), chemical softness (S = 1/2η), chemical potential (μ = (E_HOMO_ + E_LUMO_)/2), maximum charge transferred ($$\Delta N_{max} = - {\raise0.7ex\hbox{$\mu $} \!\mathord{\left/ {\vphantom {\mu \eta }}\right.\kern-0pt} \!\lower0.7ex\hbox{$\eta $}}$$), and charge transfer based on electrophilicity ($$ECT = \left( {{\Delta }N_{max} } \right)_{Nanoring} - \left( {{\Delta }N_{max} } \right)_{Complex}$$) were computationally studied to evaluate their reactivity in the presence/absence of indole. In these equations, E_HOMO_ is the energy of the frontier orbitals HOMO (highest occupied molecular orbital), and E_LUMO_ is equivalent to the energy of the frontier orbitals LUMO (lowest unoccupied molecular orbital). Also, if ECT is negative, indole behaves as an electron donor^23,24^.

The cohesive energy (E_Coh_) of each nanoring structure was calculated using Eq. (1).

1 $${\mathrm{E}}_{{{\mathrm{Coh}}}} = ({\mathrm{E}}_{{{\mathrm{total}}}} - \sum_{{{\mathrm{niEi}}}} )/{\text{ N}}$$

where E_Total_ is the total energy of the optimized nanoring structure, E_i_ is the energy of the isolated atom i (C, B, Al, or N), ni is the number of atoms of type i in the structure, and N = ∑i_ni_ is the total number of atoms in the nanoring^25^.

The adsorption energy (Eads), recovery time ($\tau$), and electrical conductivity (${\upsigma }$) were computationally studied to evaluate the sensing mechanism using Eqs. (2–4).

2 $$E_{ads} = E_{Complex} - \left( {E_{Nanoring} + E_{Indole} } \right) + E_{BSSE}$$

3 $$\tau = V_{0}^{ - 1} \times \exp \left( { - \frac{{E_{ads} }}{{k_{B} T}}} \right)$$

4 $$\sigma = AT^{3/2} e^{{\left( { - HLG/2KT} \right)}}$$

The adsorption energy (E_ads_, Eq. 2) quantifies the interaction strength between indole and the nanoring and is calculated as the difference between the total energy of the nanoring-indole complex (E_Complex_) and the sum of the energies of the isolated nanoring (E_Nanoring_) and indole (E_Indole_), with a correction for basis set superposition error (E_BSSE_) to improve accuracy^25^. The recovery time (τ, Eq. 3) represents the desorption rate of indole from the nanoring surface. It is estimated using the attempt frequency *V*_0_ (10^12^ s^−1^) and the Boltzmann factor in room temperature (T = 298 k)^26^. The electrical conductivity (σ, Eq. 4) of the nanoring is related to its electronic structure and temperature. Here, A is Richardson’s constant (6 × 10^5^ A m^−2^). The equation indicates that a smaller energy gap leads to higher conductivity, which is important for evaluating the nanoring’s sensitivity as a sensor^27^.

## Results and discussion

### Adsorption geometries and interaction energies

**Figure 2:**
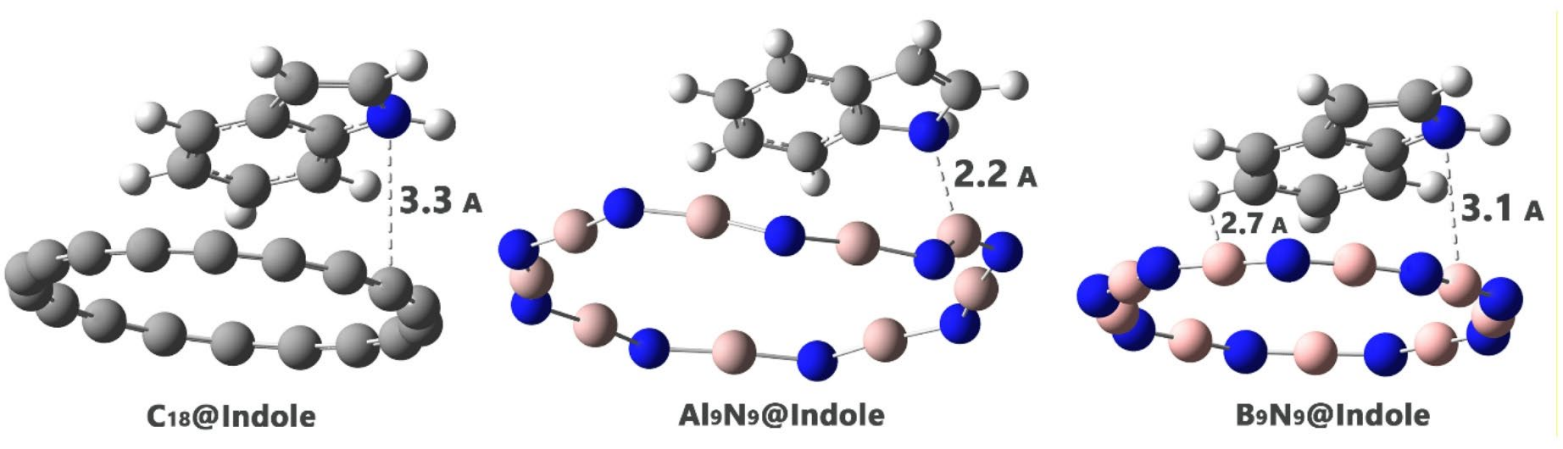
Optimized structure of each of the complexes designed in this work.

Investigating adsorption geometries and interaction energies is crucial for designing sensor-analyte complexes, as they directly influence the sensor’s sensitivity, selectivity, and performance. The adsorption geometry determines how the analyte interacts with the sensor’s surface, affecting factors such as binding strength and orientation^28^. In this regard, to achieve the most stable adsorption geometry between indole and each nanoring, indole was placed in neutral conditions (2.5 Å distance) in the vicinity of each nanoring, and then geometric optimization was performed. The optimal adsorption geometry of each designed complex is shown in Fig. 2.

A closer inspection of the optimized adsorption geometries (Fig. 2) reveals distinct interaction configurations for each nanoring. In the C_18_@Indole complex, the indole ring lies approximately parallel to the C_18_ surface, forming a weak π-π/dispersion-dominated stacking arrangement, consistent with the long intermolecular distance (3.3 Å). In B_9_N_9_@Indole, indole adopts a skewed orientation in which the pyrrole moiety approaches the electron-deficient B-N plane, enabling π-plane interactions. In Al_9_N_9_@Indole, the Indole molecule is oriented with the pyrrole nitrogen towards the Al-N ring, creating a complex interaction motif involving the → σ/π* electron pair. These geometric features explain the trend in adsorption energies: C_18_ stabilizes indole mainly through weak van der Waals forces, B_9_N_9_ benefits from reinforced π-surface and donor–acceptor interactions, and Al_9_N_9_ forms short-range, partially polarization-driven contacts. As later confirmed by NCI, QTAIM, and NBO analyses, these interactions make the dominant stabilizing contributions to the observed adsorption strengths.

However, although Al_9_N_9_@Indole has the shortest bond length (2.2 Å), this does not automatically guarantee that the interaction between indole and the Al_9_N_9_ nanoring is the strongest. The strength of the interaction is not only determined by the bond length but also by the nature of the chemical bonding and the interaction energies involved. The adsorption energy, which quantifies the overall stability of the complex, is a crucial factor in determining the strength of the interaction. Even with a shorter bond length, factors such as charge distribution, the sensor’s electronic properties, and the potential for chemical interactions between the indole and the sensor may influence the interaction strength. To accurately assess which complex has the strongest adsorption, it is necessary to examine various interaction energies, including adsorption energy, Gibbs adsorption energy, adsorption enthalpy, zero-point energy, and heat capacity. Each of these parameters plays a critical role in understanding the adsorption process and the stability of the sensor-analyte complex.

Adsorption Energy is the most direct measure of the strength of the interaction between the adsorbate (indole) and the adsorbent (C_18_, Al_9_N_9_, or B_9_N_9_). It represents the energy released or absorbed during adsorption. A more negative adsorption energy typically indicates a stronger, more stable adsorption^29^. Gibbs Adsorption Energy (∆G_ads_) accounts for the temperature and pressure conditions under which adsorption occurs. It combines the enthalpy and entropy changes associated with the adsorption process and is particularly useful for predicting the spontaneity of the adsorption at different temperatures. A negative ∆G_ads_ indicates that the adsorption is thermodynamically favorable, meaning it will occur spontaneously under given conditions^30^. Adsorption Enthalpy (∆H_ads_) is the heat released or absorbed during adsorption. It provides information about the energetic stability of the adsorption process. A negative ∆H_ads_ indicates an exothermic process, suggesting that the complex is stable and that adsorption is energetically favorable^31^. Zero-point energy (ZPE) is the lowest possible energy a system can have, even at absolute zero temperature. It is important because it accounts for quantum–mechanical effects that influence the vibrational states of the molecules involved in the adsorption process. Zero-point energy corrections can affect the overall stability of the adsorption complex, especially at very low temperatures^32^. Heat Capacity (Cv) is the amount of heat required to raise the system’s temperature by a given amount. It provides insights into the system’s thermodynamic behavior as temperature changes. The heat capacity is important for understanding how temperature affects the stability of the adsorbed complex and can provide an indication of the complex’s thermal stability and reactivity^33^. Together, these interaction energies provide a comprehensive picture of the adsorption process, enabling a better understanding of which complex has the strongest and most stable adsorption. Each of these parameters was calculated for the designed complexes (C_18_@Indole, Al_9_N_9_@Indole, and B_9_N_9_@Indole), and the results were reported in Table 1.

**Table 1:** Adsorption properties and thermodynamic parameters for the complexes of indole with C18, B9N9, and Al9N9 sensors, including adsorption energy (Eads), Gibbs free energy (∆G), enthalpy (∆H), heat capacity (Cv), and zero-point energy (ZPE).

Structure	E_ads_ (kcal/mol)	∆G_ads_ (kJ/mol)	∆H_ads_ (kJ/mol)	Cv (cal/mol-kelvin)	ZPE (Hartree)
C_18_@Indole	− 8.71	− 14.75	− 28.88	92.230	0.211
B_9_N_9_@Indole	− 20.71	− 6.66	− 56.91	91.548	0.206
Al_9_N_9_@Indole	− 9.08	− 10.50	− 64.58	106.611	0.173

According to the results, all three complexes exhibit negative adsorption energies, indicating that the adsorption process is exothermic and that energy is released during the adsorption of indole. Among the three complexes, B_9_N_9_@Indole has the strongest interaction, with an adsorption energy of − 20.71 kcal/mol, followed by Al_9_N_9_@Indole at − 9.08 kcal/mol, and C_18_@Indole at − 8.71 kcal/mol, which indicates the weakest interaction. This pattern shows that the B_9_N_9_ nanoring exhibits the strongest binding to indole, whereas the carbon-based C_18_ shows the lowest interaction. Also, all three complexes have negative ∆G_ads_ values, confirming that indole adsorption is spontaneous and thermodynamically favorable for all three systems. The enthalpy change (∆H_ads_) reflects the heat released during adsorption. Al_9_N_9_@Indole has the most exothermic ∆H at − 64.58 kJ/mol, indicating that the adsorption of indole on Al_9_N_9_ is the most strongly enthalpically favored. B_9_N_9_@Indole shows a somewhat smaller exothermic enthalpy of − 56.91 kJ/mol, while C_18_@Indole has the least negative enthalpy at − 28.88 kJ/mol, suggesting that the enthalpic contribution to adsorption is weakest in this complex. The stronger exothermicity of Al_9_N_9_ and B_9_N_9_ indicates that these sensors provide a more stable adsorption environment for indole. The adsorption energies reported in Table 1 arise directly from the combination of π-surface interactions, polarization effects, and localized donor–acceptor contributions elucidated by the structural, NCI, QTAIM, and NBO analyses.

The heat capacities (Cv) of all three complexes show only minor differences. C_18_@Indole has a Cv value of 92.230 cal/mol-K, while B9N9@Indole has 91.548 cal/mol-K, and Al_9_N_9_@Indole has 106.611 cal/mol-K. Despite these numerical differences, the variation in heat capacity is relatively small, indicating that all three systems have comparable thermal responses. This suggests that, in terms of thermal energy absorption with temperature changes, the three complexes behave similarly, with no significant difference in the amount of energy required to raise their temperature by 1 K. Thus, while Al9N9@Indole has a slightly higher Cv, the difference is not significant enough to significantly impact the thermal stability or overall heat behavior of the complexes. Finally, the zero-point energy (ZPE), which accounts for the quantum mechanical energy of the system at its lowest energy state, is lowest for Al_9_N_9_@Indole at 0.173 Hartree, followed by B_9_N_9_@Indole at 0.206 Hartree, and C18@Indole at 0.211 Hartree. A lower ZPE indicates a more stable quantum state for Al_9_N_9_@Indole, further suggesting that this complex is more thermodynamically stable.

Also, to further validate that the optimized adsorption complexes correspond to genuine minima on the potential energy surface, we analyzed the first (lowest) vibrational frequency for each nanoring-indole system. The obtained values are 15.821 cm^−1^ for C_18_@Indole, 23.260 cm^−1^ for B_9_N_9_@Indole, and 8.880 cm^−1^ for Al9N9@Indole. All three frequencies are positive, and no imaginary modes are present, confirming that the reported geometries are true local minima rather than transition states. The relatively low values, particularly for Al_9_N_9_@Indole, indicate the presence of soft vibrational modes associated with weak, large-amplitude motions such as slight tilting or sliding of indole over the nanoring surface. This behaviour is typical of non-covalent adsorption complexes, where shallow regions of the potential energy surface coexist with overall structural stability. The somewhat higher first frequency of B9N9@Indole (23.260 cm^−1^) is consistent with its stronger adsorption energy and more confined binding pocket, which restricts the motion of indole to a greater extent. Also, a complete vibrational analysis of the Al₉N₉@Indole complex yields a lowest positive frequency of 8.88 cm^−1^ with no imaginary modes, which verifies that the structure used throughout this work is at least a genuine local minimum. At the same time, we do not claim to have exhaustively proven it to be the global minimum; it is the most stable adsorption geometry identified in our calculations and serves as the basis for subsequent electronic-structure and sensing analyses. Collectively, these vibrational data corroborate our energetic and topological results.

To evaluate the sensing properties of the designed sensors, recovery time and electrical conductivity were measured. Recovery time is the time required for the sensor to return to its baseline state after detecting the analyte (indole), which is crucial for determining the sensor’s responsiveness and its ability to reset for subsequent measurements quickly. Electrical conductivity is a key parameter because it indicates how the adsorption of indole affects the flow of charge through the sensor material^26^. Electrical conductivity measures how the sensor’s electronic structure changes upon adsorption, serving as the primary measurable signal in many nanosensors. Each parameter was calculated, and the results were reported in Table 2.

**Table 2:** Adsorption energy (Eads), electrical conductivity ($\sigma$) and recovery time ($\tau$) values in each of the studied structures.

Structure	$\tau$(s)	($\sigma$) (S/m)
C_18_	–	2.95 × 10^9^
B_9_N_9_	–	2.81 × 10^9^
Al_9_N_9_	–	2.94 × 10^9^
C_18_@Indole	2.45 × 10^–6^	3.01 × 10^9^
B_9_N_9_@Indole	1.54 × 10^3^	2.88 × 10^9^
Al_9_N_9_@Indole	4.57 × 10^–6^	2.95 × 10^9^

In the case of C_18_, the conductivity increases slightly from 2.95 × 10^9^ to 3.01 × 10^9^ S/m upon adsorption of indole. This change, although positive, is relatively small, suggesting a weak interaction and limited responsiveness of C_18_ to indole. B_9_N_9_ shows a conductivity of 2.81 × 10^9^ S/m before adsorption. After indole adsorption (B_9_N_9_@Indole), the conductivity increases to 2.88 × 10^9^ S/m, which indicates a moderate interaction with the analyte. This change is significant enough to suggest that B_9_N_9_ is sensitive to indole. Al_9_N_9_ shows almost no change in conductivity, with values remaining at 2.95 × 10^9^ S/m before and after indole adsorption. This indicates that the interaction between indole and Al_9_N_9_ is minimal, making this sensor less responsive to indole compared to B_9_N_9_ and C_18_.

**Figure 3:**
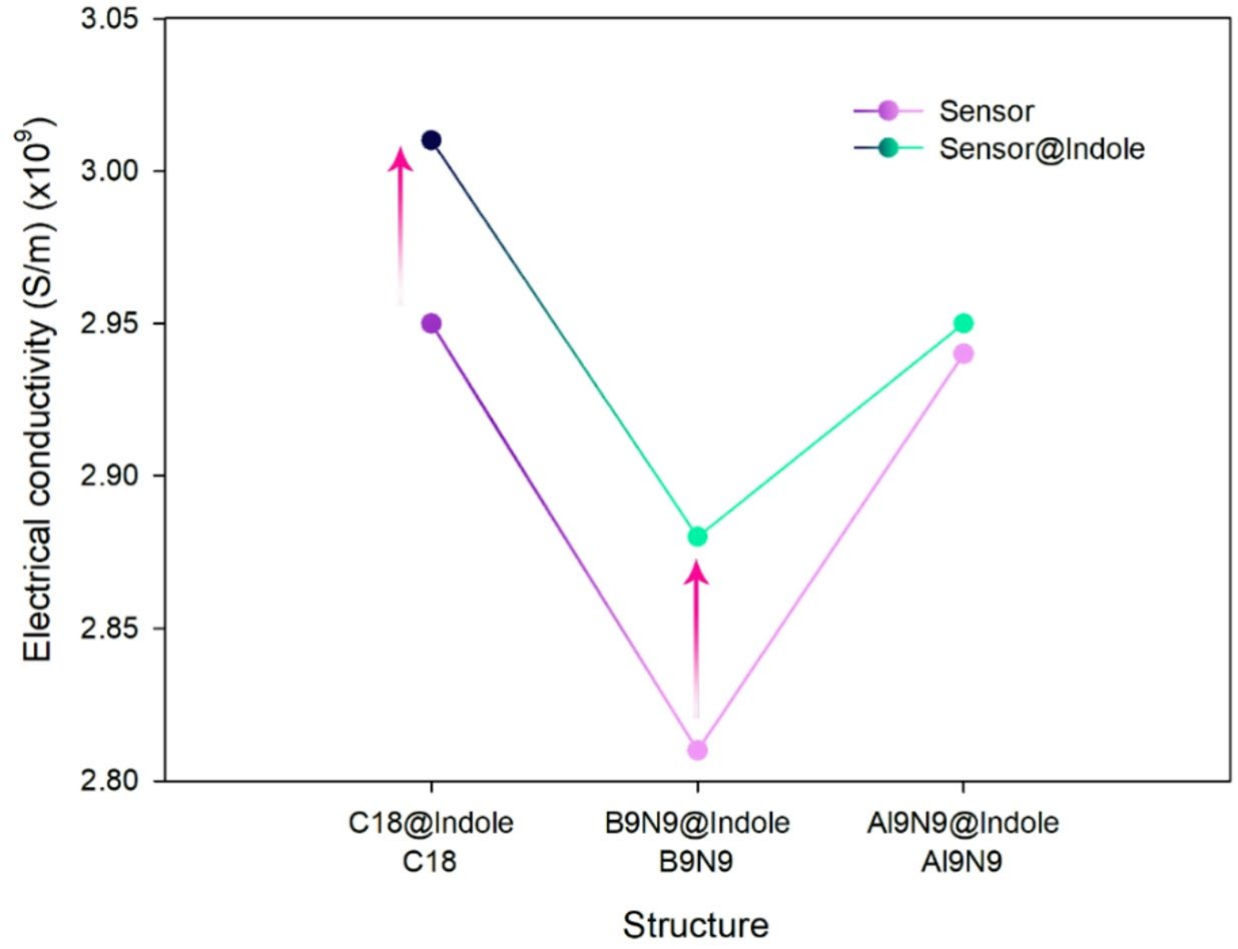
The trend of changes in electrical conductivity in the presence and absence of indole.

C_18_@Indole has a very short recovery time of 2.45 × 10^–6^ s, which is exceptionally fast. This suggests that the sensor is highly responsive and can quickly reset after interaction with indole, making it suitable for rapid detection applications. B9N9@Indole has a much longer recovery time of 1.54 × 10^3^ s, indicating that, while the sensor interacts strongly with indole, it takes longer to return to its baseline state. This longer recovery time could be a limitation for applications requiring quick successive measurements, but does not detract from its overall sensing capability. Al_9_N_9_@Indole has a recovery time of 4.57 × 10^–6^ s, which is quite fast, comparable to that of C_18_. However, as mentioned earlier, the minimal change in conductivity for Al_9_N_9_@Indole suggests that this fast recovery time may not compensate for its weak responsiveness to indole (Fig. 3).

In the study, B_9_N_9_ is identified as the best electrochemical sensor for single-use applications due to its strong interaction with indole and its ability to induce significant changes in conductivity. It appears that the adsorption of indole onto B_9_N_9_, driven by noncovalent interactions such as van der Waals forces and π-π stacking, redistributes electron density within the B_9_N_9_ nanoring, ultimately leading to charge transfer between indole and the sensor. The adsorption of indole redistributes electron density within the B_9_N_9_ nanoring, leading to charge transfer between indole and the sensor. This electron donation from indole via its HOMO to the LUMO of the B_9_N_9_ nanoring (as predicted and studied in detail by ECT analysis in "Electronic properties analyses" section) appears to alter the sensor’s electronic structure, leading to an increase in conductivity. This conductivity change, measurable through the sensor, provides the basis for sensing indole. Despite the long recovery time, which means it takes a while for the sensor to return to its baseline after detecting indole, this slower recovery is acceptable for single-use electrochemical sensing, where only one measurement is needed per use.

On the other hand, C_18_ can act as an electrochemical sensor, but with lower accuracy than B_9_N_9_. The adsorption energy of C_18_@Indole is much lower, suggesting that the interaction between C_18_ and indole is weaker. This weaker interaction results in a smaller change in conductivity, a less significant response than with B_9_N_9_. As a result, C_18_ is less sensitive and accurate as an electrochemical sensor. The lower adsorption strength and smaller change in conductivity mean that C_18_ is not as reliable for precise measurements, limiting its potential for high-accuracy sensing applications. In contrast, Al_9_N_9_ shows no appreciable change in conductivity in the presence of indole. The conductivity of Al_9_N_9_ remains unchanged before and after indole adsorption, indicating that Al_9_N_9_ does not interact effectively with indole in a manner that alters its electronic properties or conductivity. This lack of response makes Al_9_N_9_ unsuitable for electrochemical sensing, as it fails to produce a detectable signal upon exposure to indole.

### NCI and QTAIM analysis

**Figure 4:**
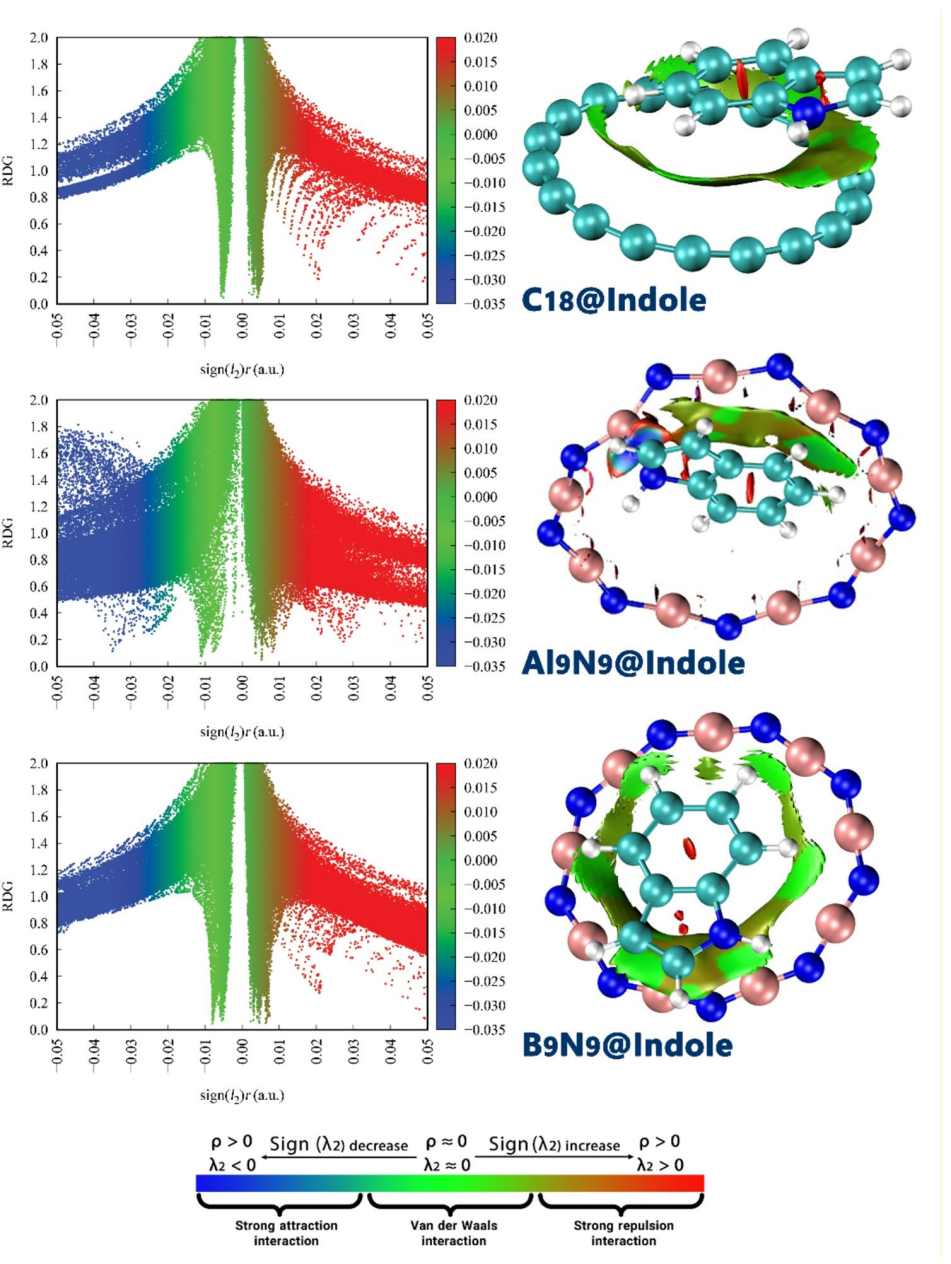
NCI and RDG contours in C_18_, B_9_N_9_ and Al_9_N_9_ complexes with indole.

The examination of Non-Covalent Interaction (NCI) regions and Reduced Density Gradient (RDG) contours is of paramount importance in the rational design of molecular complexes, as it provides a direct, visual map of the attractive and repulsive forces that govern molecular association. Unlike other methods, NCI-RDG analysis allows for the unambiguous identification and spatial localization of various non-covalent interactions (such as hydrogen bonding, van der Waals forces, and steric repulsion) based on the electron density and its derivatives. This is important not only for understanding the strength, but also for understanding the nature of the binding, and allows for examining the stability, selectivity, and overall feasibility of a complex^34^. The RDG and NCI contours for each of the designed complexes are shown in Fig. 4.

The RDG plot for B_9_N_9_@Indole displays a prominent region of strong attraction (blue), with a significant negative value for sign(∇ρ), indicating the most significant and favorable interaction between B9N9 and indole, driven by non-covalent forces, excluding electrostatic interactions, as indicated by the absence of direct electrostatic contributions in the QTAIM and NCI analysis.

The RDG plot for Al_9_N_9_@Indole also shows regions of negative sign(∇ρ) (blue), indicating attraction, but the peaks are less pronounced compared to B_9_N_9_. The plot also shows a broader distribution in the positive region, suggesting weaker interactions or areas of van der Waals forces. The NCI contours confirm this, as the interaction between Al_9_N_9_ and indole is weaker and more spatially spread out. While there is still attractive interaction, it is not as intense or localized as in B_9_N_9_, indicating a moderate level of binding between Al_9_N_9_ and indole. The RDG plot for C_18_@Indole shows the smallest shift towards the blue (attractive) region and a higher distribution towards the red (repulsive) area. This suggests that, although an attractive interaction is present, C_18_ exhibits the weakest interaction with indole among B_9_N_9_, Al_9_N_9_, and C_18_. The NCI contour plot also shows less pronounced blue areas, with more regions of green and red, indicating weaker and less stable interactions. The overall distribution suggests that the binding between C_18_ and indole is less favorable, with a significant portion of the interaction characterized by repulsive forces or very weak attraction. These results agree with the calculated adsorption energy.

We acknowledge that the designed complexes do not exhibit electrostatic interactions, as evidenced by the absence of significant electrostatic forces in our QTAIM and NCI analysis. The interaction between indole and the nanorings is primarily driven by non-covalent forces such as van der Waals interactions and other weak intermolecular forces, but electrostatic interactions were not observed to play a major role in the complex formation.

**Figure 5:**
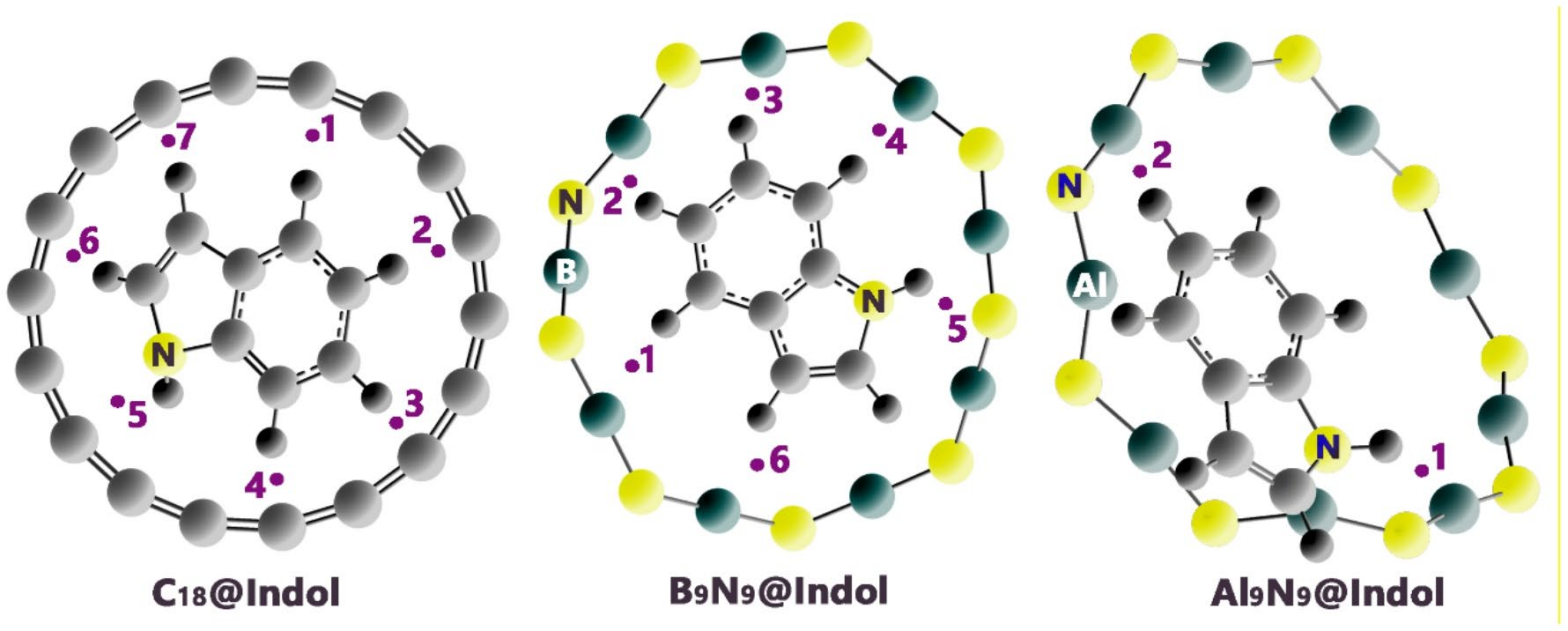
Location of BCP in each of the complexes studied in this work.

To further validate the results obtained from the NCI and RDG contours, Quantum Theory of Atoms in Molecules (QTAIM) analysis was performed. Quantum Theory of Atoms in Molecules (QTAIM) is a fundamental tool in computational chemistry for rigorously investigating the properties of atoms and the nature of chemical bonds within a molecule or complex. Its power lies in its ability to define bonding interactions topologically through the analysis of the electron density, ρ(r)^35^. Central to this analysis is the concept of the Bond Critical Point (BCP), a point between two nuclei where the electron density is a minimum along the bond path and a maximum in the perpendicular directions (Fig. 5). The electronic properties calculated at this BCP provide deep insight into the bond’s character. Key parameters include the electron density itself, ρ(r), which indicates the shared charge concentration; its Laplacian, ∇^2^ρ(r), which reveals whether the density is locally depleted (∇^2^ρ(r) > 0, closed-shell interactions like ionic or van der Waals) or concentrated (∇^2^ρ(r) < 0, shared interactions like covalent bonds); the kinetic energy density, G(r); the potential energy density, V(r); and the total electron energy density, Hb = V(r) + G(r), where a negative H(r) signifies a stabilizing interaction^36^.

To precisely categorize hydrogen bonds and other non-covalent interactions, the classification proposed by Rozas et al. is highly valuable. According to this framework, the nature of a bond is determined by the synergy between the Laplacian ∇^2^ρ(r) and the total electron energy density Hb at the BCP. A negative H(r) coupled with a negative ∇^2^ρ(r) indicates a strong, covalent-like hydrogen bond, characterized by significant electron sharing. A positive Hb with a negative ∇^2^ρ(r) suggests a medium-strength hydrogen bond. Finally, when both H(r) and ∇^2^ρ(r) are positive, the interaction is classified as weak and is primarily governed by van der Waals forces^37,38^. By applying this classification to the identified BCPs between the nanoring and indole, the strength and fundamental nature of the stabilizing interaction in each of the designed complexes can be quantitatively assessed and an accurate measure can be provided to explain their relative stability and electronic behaviors.

The parameters provided in Table 3 (electron density (ρ(r)), Laplacian of electron density (∇^2^ρ(r)), kinetic energy density (G(r)), potential energy density (V(r)), and the total electron energy density (H(r))) help us assess the nature and strength of these interactions in different molecular structures.

**Table 3:** The obtained values for ρ(r), ∇^2^ρ(r), V(r), G(r), Hb and the VIR in the BCP.

Structure	BCP	ρ(r)	∇^2^ρ(r)	G(r)	V(r)	VIR	Hb
C_18_@Indole	1	0.00564	− 0.0048	0.00369	− 0.00107	0.00262	0.00262
	2	0.00554	− 0.0045	0.00360	− 0.00091	0.00268	0.00268
	3	0.00567	− 0.0046	0.00368	− 0.00095	0.00272	0.00273
	4	0.00505	− 0.0042	0.00323	− 0.00095	0.00227	0.00227
	5	0.00585	− 0.0046	0.00380	− 0.00077	0.00303	0.00302
	6	0.00567	− 0.0040	0.00325	− 0.00075	0.00249	0.00250
	7	0.00505	− 0.0042	0.00320	− 0.00095	0.00228	0.00225
B_9_N_9_@Indole	1	0.00528	− 0.00426	0.00331	− 0.00094	0.00237	0.00236
	2	0.00584	− 0.00441	0.00364	− 0.00077	0.00286	0.00287
	3	0.00559	− 0.00424	0.00349	− 0.00079	0.00274	0.00271
	4	0.00597	− 0.00464	0.00373	− 0.00091	0.00283	0.00283
	5	0.00811	− 0.00554	0.00498	− 0.00056	0.00442	0.00442
	6	0.00805	− 0.00505	0.00434	− 0.00068	0.00367	0.00367
Al_9_N_9_@Indole	1	0.03607	− 0.03438	0.03718	0.00281	0.03999	0.03998
	2	0.01125	0.01125	0.00406	0.00048	0.00454	0.00454

For each structure (C_18_@Indole, B_9_N_9_@Indole, and Al_9_N_9_@Indole), the provided BCP values offer a detailed look at how electron density behaves in the context of chemical bonding. A close analysis of the values for each molecule shows how the various parameters interact to characterize the bond type.

Starting with the C_18_@Indole structure, the values of electron density (ρ(r)) range from 0.00505 to 0.00585, all of which indicate low electron density at the BCP, suggesting weak to moderate bonding interactions. The Laplacian values (∇^2^ρ(r)) are negative across all BCPs, which is characteristic of shared interactions (covalent bonds). The kinetic energy density (G(r)) is consistently positive, while the potential energy density (V(r)) is negative, resulting in total electron energy densities (H(r)) that are mostly negative. This suggests stabilizing interactions, particularly those with stronger electron sharing, indicative of covalent-like bonds. The VIR (Variation in Electron Density, which could indicate the stability or interaction strength) is relatively consistent, with values of approximately 0.0025 to 0.003, supporting the notion of moderate bonding strength. The Hb values, ranging from 0.00227 to 0.00302, reflect similar conclusions regarding the overall strength of these interactions, with no extreme variation in the hydrogen bond strength.

Moving to the B9N9@Indole structure, we observe slightly higher electron density values at the BCPs, ranging from 0.00528 to 0.00811. This suggests that the interactions between B9N9 and Indole are somewhat stronger than those between C^18^@Indole and Indole. Notably, the Laplacian values (∇^2^ρ(r)) remain negative for most BCPs, though there is an increase in their magnitude, which may reflect slightly more concentrated electron density at these points. The total energy densities (H(r)) are mostly negative, with a few cases where they are slightly positive, indicating covalent-like bonding. The VIR values for this structure are generally higher than those in C_18_@Indole, peaking at 0.00442, suggesting stronger or more varied bonding interactions. The Hb values also show an increase compared to C_18_@Indole, reaching 0.00442, further supporting the idea of stronger bonding interactions.

Lastly, in the Al_9_N_9_@Indole structure, the values of electron density at the BCPs are significantly higher, particularly in BCP1, where ρ(r) reaches 0.03607. This is indicative of very strong bonding interactions, likely involving considerable electron sharing. The Laplacian values (∇^2^ρ(r)) are notably more negative, with a drastic shift in the first BCP to a value of − 0.03438, suggesting a highly concentrated electron density and covalent-like bonding. This concentration of electron density is further confirmed by the total electron energy density (H(r)), which is negative, indicating a stabilizing interaction. The VIR value for the first BCP is very high at 0.03999, reflecting the significant strength of the interaction. The hydrogen bond strength (Hb) in this structure is also the highest observed, reaching 0.03998 at BCP1, which is consistent with the very strong, highly covalent-like bond suggested by the other parameters.

Finally, the analysis of the data reveals that the Al_9_N_9_@Indole complex exhibits the strongest and most covalent-like interactions, as evidenced by the higher electron density, more negative Laplacian, and the negative total electron energy density. This is followed by the B_9_N_9_@Indole structure, which shows moderate-to-strong bonding interactions with a mix of covalent and electrostatic characteristics. Finally, the C_18_@Indole complex displays the weakest interactions, with electron density values indicating relatively weak bonds, mostly of a covalent nature, though still stabilizing in character.

While the adsorption energy suggests B_9_N_9_ forms the most stable complex overall, the QTAIM analysis reveals that Al_9_N_9_@Indole features specific bond paths with a covalent character and electron density concentration that surpasses those found in the other complexes. This detailed QTAIM analysis provides crucial insights into the bonding nature and relative stability of these molecular systems, with clear distinctions in the strength and type of interactions across the different complexes. This detailed QTAIM analysis provides important insights into the nature of bonding and the relative stability of these molecular systems, with clear distinctions in the strength and type of interactions in different complexes, and is consistent with reported values for adsorption energy (reported in Table 1).

### Electronic properties analyses

The most important electronic properties, including reactivity parameters (such as the energy of HOMO/LUMO frontier orbitals and their gaps, chemical hardness, chemical softness, chemical potential, and maximum charge transfer), DOS, and NBO were studied. Reactivity parameters describe how a molecule responds electronically during chemical interactions. A smaller HLG and higher softness generally indicate greater reactivity, while hardness and chemical potential help predict stability and the direction of electron flow^33^. Complementing these parameters, charge-transfer parameters, such as ΔNmax, estimate the maximum number of electrons a system can accept or donate, and electrophilic charge transfer (ECT) quantifies the direction of electron flow between interacting species^39,40^. In this regard, each parameter was calculated, and the results were reported in Table 4.

**Table 4:** Reactivity and load transfer parameters for each of the designed structures.

Structure	LUMO (eV)	HOMO (eV)	HLG (eV)	η (eV)	μ (eV)	S (eV^−1^)	∆N_max_	ECT
C_18_	− 3.76	− 5.97	2.21	1.10	− 4.86	0.45	4.41	–
B_9_N_9_	− 1.71	− 6.48	4.74	2.38	− 4.09	0.21	1.71	–
Al_9_N_9_	− 3.01	− 5.44	2.43	1.21	− 4.22	0.41	3.48	–
C_18_@Indole	− 3.72	− 4.94	1.22	0.61	− 4.33	0.81	7.09	− 2.68
B_9_N_9_@Indole	− 1.72	− 5.13	3.41	1.70	− 3.42	0.29	2.01	− 0.31
Al_9_N_9_@Indole	− 2.92	− 5.15	2.23	1.11	− 4.03	0.44	3.63	− 0.15

**Figure 6:**
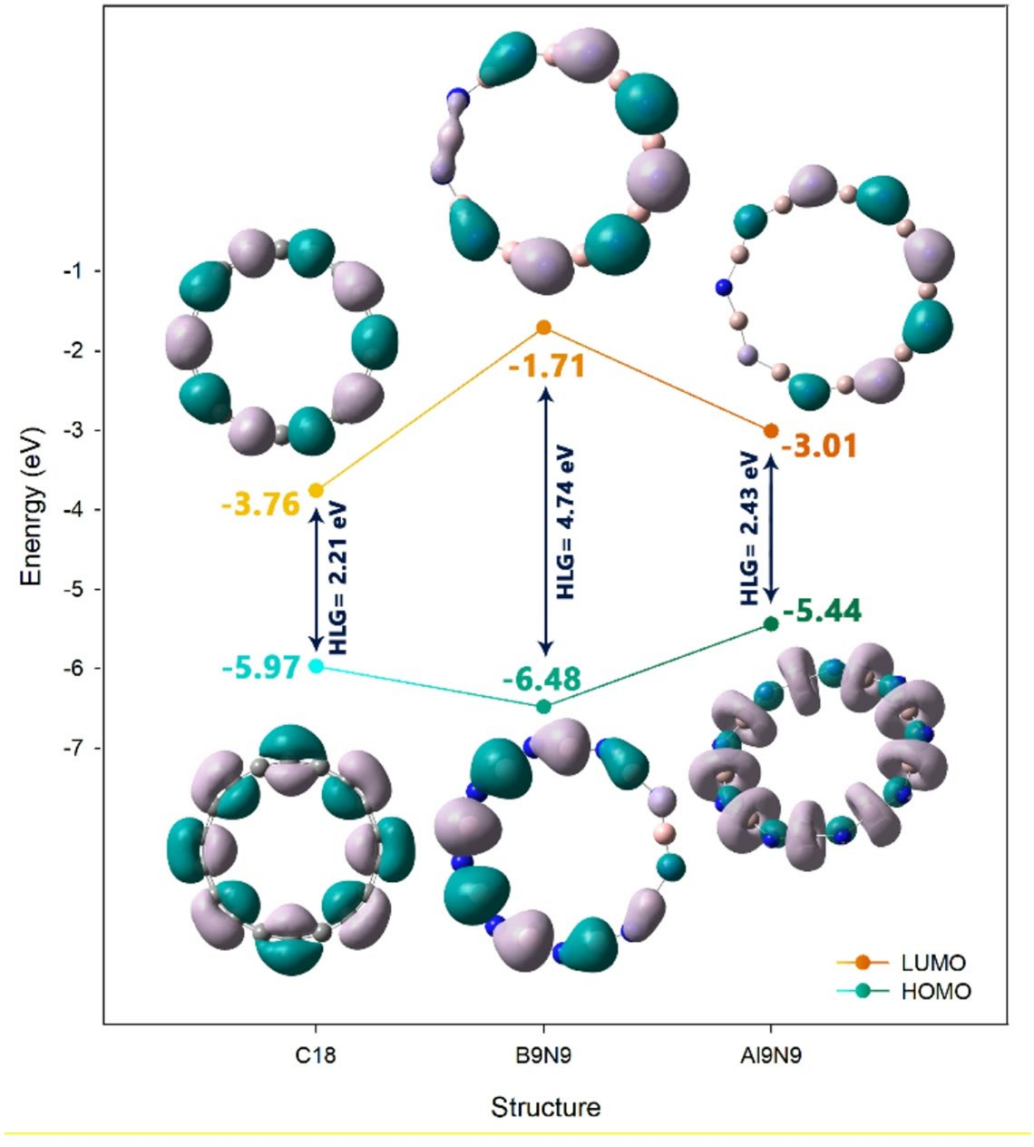
The spatial shape and energy of the HOMO and LUMO orbitals in each of the nanorings studied.

The analysis of the reactivity and load transfer parameters for the C_18_, B_9_N_9_, and Al_9_N_9_ structures, both in their isolated states and in complex with Indole, reveals significant insights into their electronic properties and the nature of their interaction with the adsorbate. In the absence of Indole, the three structures exhibit distinct electronic profiles. The HOMO–LUMO Gap (HLG), a key indicator of kinetic stability, is largest for B_9_N_9_ (4.74 eV), suggesting it is the most stable and least reactive of the three. In contrast, C_18_ has the smallest HLG (2.21 eV), indicating high reactivity and low stability, while Al_9_N_9_ presents an intermediate value (2.43 eV) (See Fig. 6). This trend is directly mirrored in the chemical hardness (η), where a larger HLG corresponds to a higher η. Consequently, B_9_N_9_ is the hardest (2.38 eV) and C_18_ the softest (1.10 eV). The chemical potential (μ), which gauges the tendency of electrons to escape, shows that C18 has the highest value (− 4.86 eV), signifying the strongest driving force to accept electrons, whereas B9N9 has the lowest (− 4.09 eV). The electrophilicity index (S) and the fraction of electron transfer (∆Nmax) further confirm C_18_ as the most potent electrophile, with the highest values (S = 0.45 eV^−1^, ∆Nmax = 4.41), poised to accept a significant amount of electron density from a nucleophile.

The introduction of Indole dramatically alters the electronic landscape, with the most pronounced effect observed on the C_18_ structure. For C_18_@Indole, the HLG collapses from2.21 to 1.22 eV, and its hardness is halved from 1.10 to 0.61 eV. This indicates a substantial increase in reactivity and a drastic destabilization of the C18 ring upon adsorption. Concurrently, its electrophilicity (S) surges from 0.45 to 0.81, and its ∆Nmax increases from 4.41 to 7.09, suggesting an enhanced capacity to attract electron density from Indole.

In comparison, the changes for B_9_N_9_ and Al_9_N_9_ upon Indole adsorption are more subtle. For B_9_N_9_@Indole, the HLG decreases from 4.74 to 3.41 eV, and the hardness reduces from 2.38 to 1.70 eV, indicating a moderate increase in reactivity. Its electrophilicity and ∆Nmax see only slight increases. Similarly, for Al_9_N_9_@Indole, the changes in HLG, hardness, chemical potential, and electrophilicity are minimal. The negative ECT values for all complexes confirm that all indoles act as electron donors and the charge is transferred from indole to the nanorings.

**Figure 7:**
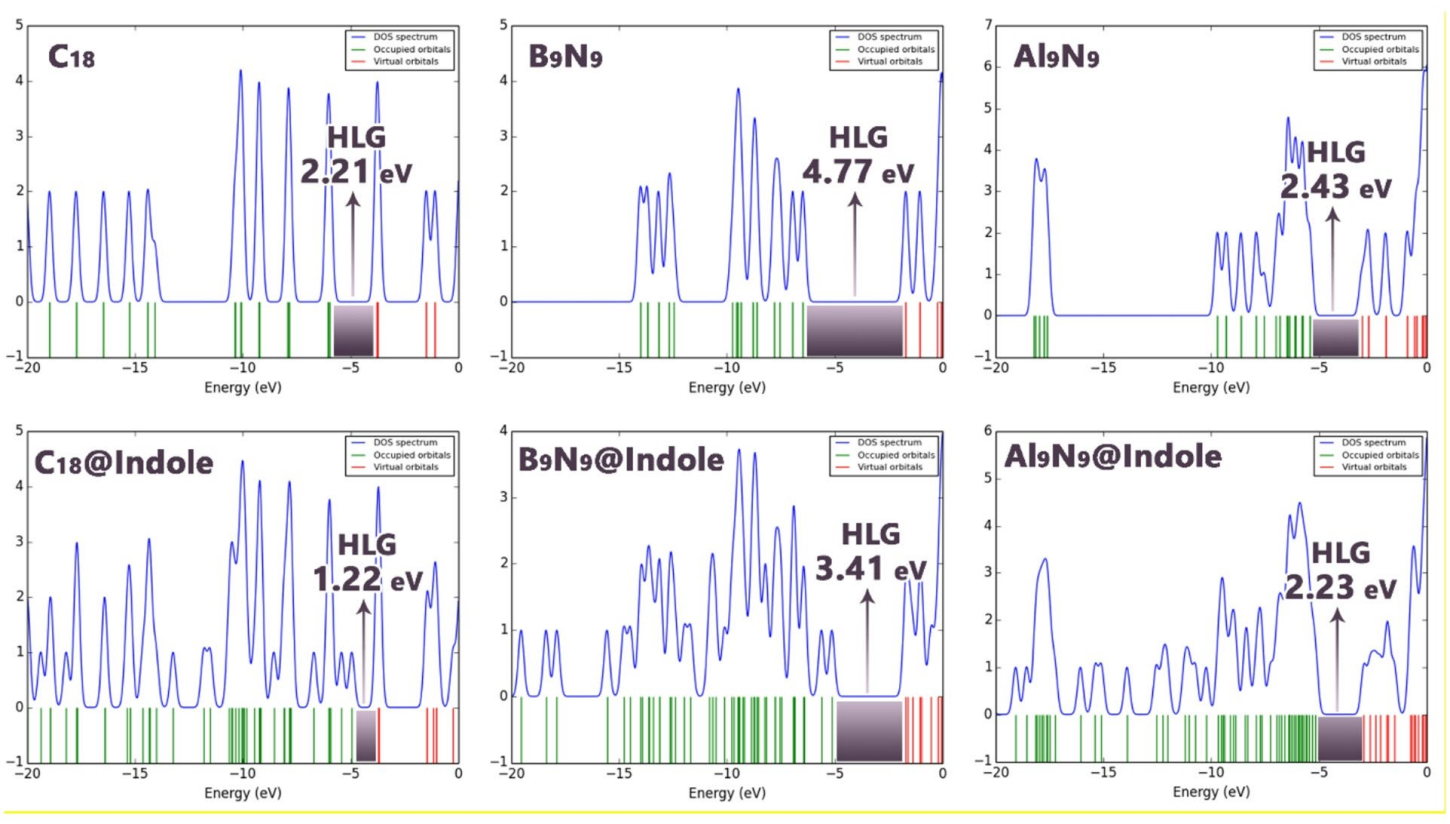
DOS plot for each of the structures designed in this work.

To gain a deeper understanding of the electronic structure changes upon complexation, the Density of States (DOS) plots for all isolated nanorings and their corresponding indole complexes were calculated and are presented in Fig. 7. The DOS analysis serves as an excellent visual complement to the quantitative orbital energy data, providing an intuitive representation of the electronic landscape and, crucially, the energy gap^41^.

The DOS plots for the pristine nanorings (C_18_, B_9_N_9_, and Al_9_N_9_) clearly reflect their distinct electronic characters. The B9N9 nanoring exhibits a wide separation between the highest occupied and lowest unoccupied states, which corresponds visually to its large HOMO–LUMO Gap (HLG) of 4.74 eV reported in Table 4. This significant gap is a hallmark of high kinetic stability. In contrast, the C_18_ nanoring shows a much narrower gap, consistent with its smaller tabulated HLG of 2.21 eV and indicative of its inherently higher chemical reactivity. The Al_9_N_9_ nanoring presents an intermediate gap, visually aligning with its calculated HLG of 2.43 eV.

The most pronounced and consistent feature observed in the DOS plots of the complexes is the notable narrowing of the energy gap compared to their parent nanorings. This visual trend directly confirms the systematic decrease in HLG values documented in Table 4. This phenomenon can be attributed to the emergence of new hybridized states near the Fermi level, resulting from the orbital interactions between indole and the nanorings.

Furthermore, the DOS plots allow for a qualitative assessment of charge transfer. The shifts in the peak positions and the changes in the shape of the curves near the frontier regions provide visual evidence of the orbital mixing that facilitates the electron donation from indole (donor) to the nanoring (acceptor), as quantitatively described by the negative ECT values in Table 4.

**Figure 8:**
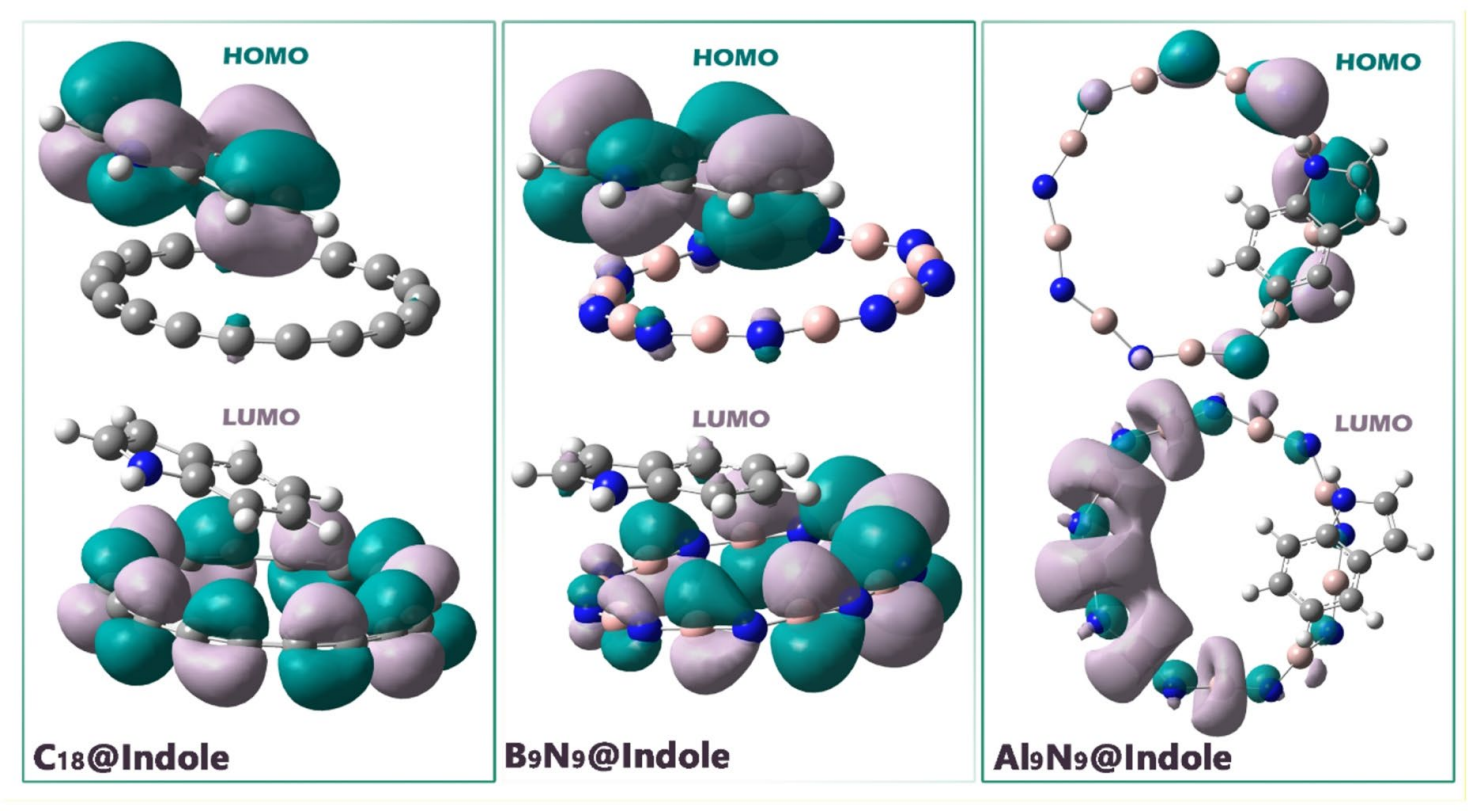
How to distribute HOMO and LUMO orbitals on designed complexes.

Investigating the spatial features of the Frontier Molecular Orbitals (FMOs), specifically the HOMO and the LUMO, is key to establishing the nature of the intermolecular complex. The positions of the FMOs speaks directly to the areas of greatest chemical reactivity, likely route for charge transfer, and the very character of the complex (charge-transfer, donor–acceptor, etc.)^42^. The spatial separation of the HOMO and LUMO between those molecules directly conveys which molecule is the electronic donor and which molecule is the electronic acceptor during an electronic transition or electronic interaction (Fig. 8).

The localization of the frontier orbitals shows that in all three complexes indole acts predominantly as the electron donor (HOMO on indole) and the nanostructure as the electron acceptor (LUMO on C18, B_9_N_9_, Al_9_N_9_), which is favorable for charge-transfer processes upon excitation; however, Al_9_N_9_@Indole has a significant additional advantage: while its LUMO remains fully localized on Al_9_N_9_, the HOMO is delocalized over both Al_9_N_9_ and indole, meaning that the initial donor orbital is electronically “bridged” to the acceptor fragment, increasing orbital overlap and electronic coupling between the two parts. In C18@Indole and B_9_N_9_@Indole, the clear spatial separation of HOMO (only on indole) and LUMO (only on the ring) supports charge transfer but also implies that the transition relies purely on long-range interaction between two distinct fragments; in contrast, the partial extension of the HOMO onto Al_9_N_9_ in Al_9_N_9_@Indole reduces the effective distance between donor and acceptor densities, facilitating more efficient charge injection into the LUMO, lowering the reorganization barrier and potentially decreasing recombination. This mixed character of the HOMO also means that any change in the electronic structure of indole (for example, due to binding, protonation, or analyte sensing) can directly modulate the frontier orbitals of the Al9N9 framework itself, making the electronic response of the whole system more sensitive and tunable. Therefore, it seems that the distribution in Al9N9@Indole combines donor → acceptor directed charge transfer (LUMO on Al_9_N_9_) with stronger electronic coupling and better coupling between the two moieties (shared HOMO), improving charge transfer, which is beneficial for sensing applications. It should be noted that this distribution pattern overlaps well with the negative values reported for ECT (in terms of the direction of charge transfer).

Natural Bond Orbital (NBO) analysis is a fundamental quantum chemical tool for understanding the molecular interactions that stabilize a complex beyond simple electrostatic forces. It provides a detailed picture of electron density distribution and identifies hyperconjugative interactions, which are crucial for explaining molecular stability, reactivity, and bonding. Within this framework, the second-order perturbation energy (E^(2)^) is a particularly important quantity, as it estimates the stabilization energy associated with the donation of electron density from a filled donor orbital (e.g., a σ or π bonding orbital, or a lone pair LP) into an empty acceptor orbital (e.g., a σ* or π* anti-bonding orbital). A higher E^(2)^ value indicates a stronger donor–acceptor interaction, signifying a more significant contribution to the overall stability of the system. Analyzing the types of electronic transitions involved further reveals the nature of these interactions, distinguishing between σ-conjugation, π-delocalization, and lone pair donations^43^.

The Natural Bond Orbital (NBO) analysis presented in Table 6 reveals significant differences in the nature and strength of intramolecular interactions within the three studied complexes, C_18_@Indole, B_9_N_9_@Indole, and Al_9_N_9_@Indole. The key metric for comparison is the second-order perturbation energy E^(2)^, which indicates the stabilization energy resulting from electron delocalization from a donor orbital to an acceptor orbital (Table 5).

**Table 5:** Calculated values of NBOs analysis for the studied complexes.

Complex	Donor (i)	Type	Acceptor (j)	Type	E^(2)^ kcal mol^−1^	E(j)-E(i) a.u	F(i,j) a.u
C_18_@Indole	C1–C2	$\sigma$	C1–C18	$\sigma^{*}$	6.74	1.33	0.085
	C3–C4	$\pi$	C1–C2	$\pi^{*}$	7.87	0.27	0.042
	N31	LP (1)	C7–C8	$\pi^{*}$	0.11	0.28	0.005
B_9_N_9_@Indole	C1–C2	$\sigma$	C3–C11	$\sigma^{*}$	5.03	1.06	0.065
	C11–C12	$\pi$	B24–N27	$\pi^{*}$	16.59	0.09	0.062
	C6	LP (1)	C1–C2	$\pi^{*}$	57.96	0.12	0.090
Al_9_N_9_@Indole	C1–C2	$\sigma$	C3–C11	$\sigma^{*}$	4.71	1.08	0.064
	Al20–N32	$\pi$	Al19–N31	$\pi^{*}$	10.14	0.24	0.044
	C3	LP (1)	C11–C12	$\pi^{*}$	37.59	0.11	0.075

Beginning with the C_18_@Indole complex, the recorded second-order stabilization energies (E^(2)^) are relatively low. The primary interactions are weak hyperconjugative stabilizations, such as a σ(C1–C2)→σ*(C1–C18) interaction with an E^(2)^ of 6.74 kcal/mol and a π(C3–C4)→π*(C1–C2) interaction at 7.87 kcal/mol. Notably, the lone pair (LP) on the indole’s nitrogen atom (N31) donates very little energy (0.11 kcal/mol) into the π* system of the carbon ring, indicating a weak non-covalent interaction between the indole and the C^18^ nanoring in this model.

In stark contrast, the B_9_N_9_@Indole complex exhibits much stronger and more consequential charge transfer. The NBO data for B_9_N_9_@Indole reveals an exceptionally strong interaction, where a lone pair (LP) on carbon C6 of the indole donates electron density into the π* antibonding orbital of the C1–C2 bond of the ring, with a very high E^(2)^ value of 57.96 kcal/mol. This represents a massive flow of electron density from the analyte to the sensor material. Furthermore, there is an additional significant π→π* back-donation from the ring’s C11–C12 bond to the B24-N27 π* orbital (E^(2)^ = 16.59 kcal/mol). This two-way, high-magnitude charge transfer creates a highly conductive pathway between the indole and the B_9_N_9_ ring. When integrated into an electrode, this efficient charge delocalization would lead to a sharp change in current, producing a strong and easily measurable electrical signal in the presence of indole, making B_9_N_9_ an excellent candidate for electrochemical sensing.

The Al_9_N_9_@Indole complex presents an intermediate electronic profile. It shows a σ→σ* interaction similar to the boron system, with an E^(2)^ of 4.71 kcal/mol. A key feature is an internal charge transfer within the Al_9_N_9_ ring itself, as seen in the π(Al20-N32)→π*(Al19-N31) interaction with an E^(2)^ of 10.14 kcal/mol. Most importantly, the interaction from a carbon lone pair (C3 LP) into a π* orbital (C11-C12) is also very strong at 37.59 kcal/mol, confirming a significant donor–acceptor mechanism similar to, though less intense than, that in the B_9_N_9_ complex. This indicates a substantial delocalization of electron density across the inorganic ring’s π-system. The adsorption of the indole molecule, while also involving a lone pair donation from C3 (E^(2)^ = 37.59 kcal/mol), primarily perturbs this pre-existing, sensitive electronic structure of the Al_9_N_9_ ring. Such a pronounced redistribution of electron density within the ring’s conjugated system is highly likely to result in a significant shift in its absorption spectrum, manifesting as an obvious color change visible to the naked eye. For this purpose, the UV–vis spectrum of each of the compounds in the presence and absence of indole were studied in detail in the next section.

### UV–Vis spectrum

Examining the UV spectrum is essential for evaluating the colorimetric capability of a sensor, as parameters such as maximum absorption wavelength (λmax) and exciton energy (Eex) directly determine the sensor’s optical response. Changes in λmax and changes in exciton energy indicate changes in electronic transitions after analyte binding, which affect the visibility and sensitivity of colorimetric detection. However, based on the previously reported adsorption energy values, the C_18_ complexes were not considered for further calculations because their positive adsorption energies indicate non-spontaneous and unstable adsorption, making them unsuitable for reliable sensing applications^44^. The data presented in Table 6 reveals significant shifts in the λmax and Eex for the C_18_, B_9_N_9_, and Al_9_N_9_ structures upon interaction with indole, providing a clear basis for comparing their suitability as colorimetric sensors.

**Table 6:** Theoretical λmax and Eex for various C_18_, B_9_N_9_, and Al_9_N_9_ nanorings and their indole complexes.

Structure	λ_max_ (nm)	E_ex_ (eV)
C_18_	527	2.34
B_9_N_9_	201	6.14
Al_9_N_9_	376	3.29
C_18_@Indole	321	3.85
B_9_N_9_@Indole	248	4.98
Al_9_N_9_@Indole	463	2.67

Analysis of the individual structures shows distinct baseline properties. The C_18_ structure has a λmax of 527 nm, which lies in the green region of the visible spectrum. In contrast, B_9_N_9_ has a very high-energy absorption in the deep ultraviolet (λmax = 201 nm), while Al_9_N_9_ absorbs in the near-UV to violet region (λmax = 376 nm). The key to an effective colorimetric sensor, however, is not its initial color but the magnitude of the visible change that occurs upon binding the target molecule.

Upon complexation with indole, all three structures exhibit a change in their optical properties. For C_18_, the λmax undergoes a substantial blue-shift of 206 nm (from 527 to 321 nm), moving its absorption from the green to the UV region. This corresponds to a significant increase in exciton energy (from 2.34 to 3.85 eV). Conversely, B_9_N_9_@Indole shows a smaller blue-shift of 47 nm, but its absorption remains firmly in the UV range (from 201 to 248 nm), making any color change invisible to the human eye. The most compelling response is observed for Al_9_N_9_, which undergoes a pronounced red-shift of 87 nm (from 376 to 463 nm). This shift moves its absorption from the violet/UV edge into the blue region of the visible spectrum.

This shift for Al_9_N_9_ is critically important for a visible colorimetric response. A material that absorbs blue light will appear to the human eye as its complementary color, which is orange/yellow. Therefore, the Al_9_N_9_ sensor would be expected to change color upon indole binding, likely from a pale, nearly colorless appearance (absorbing in the violet) to a distinct orange or yellow hue (as it now absorbs blue light at 463 nm). This change is highly perceptible.

Also, the explanation for the low ƒ value in each of the complexes in the presence of Indole is also consistent with the observation of Choudhury et al., who synthesized a zwitterionic fluorescent probe with a similarly low oscillator strength (ƒ = 0.005), but showed strong solvent-dependent color changes^45^. Thus, a lower ƒ value may lead to a significant visible optical change when the system also shows a strong spectral change under a specific environmental condition (e.g., a change in solvent), which is consistent with the background of a large color change.

**Figure 9:**
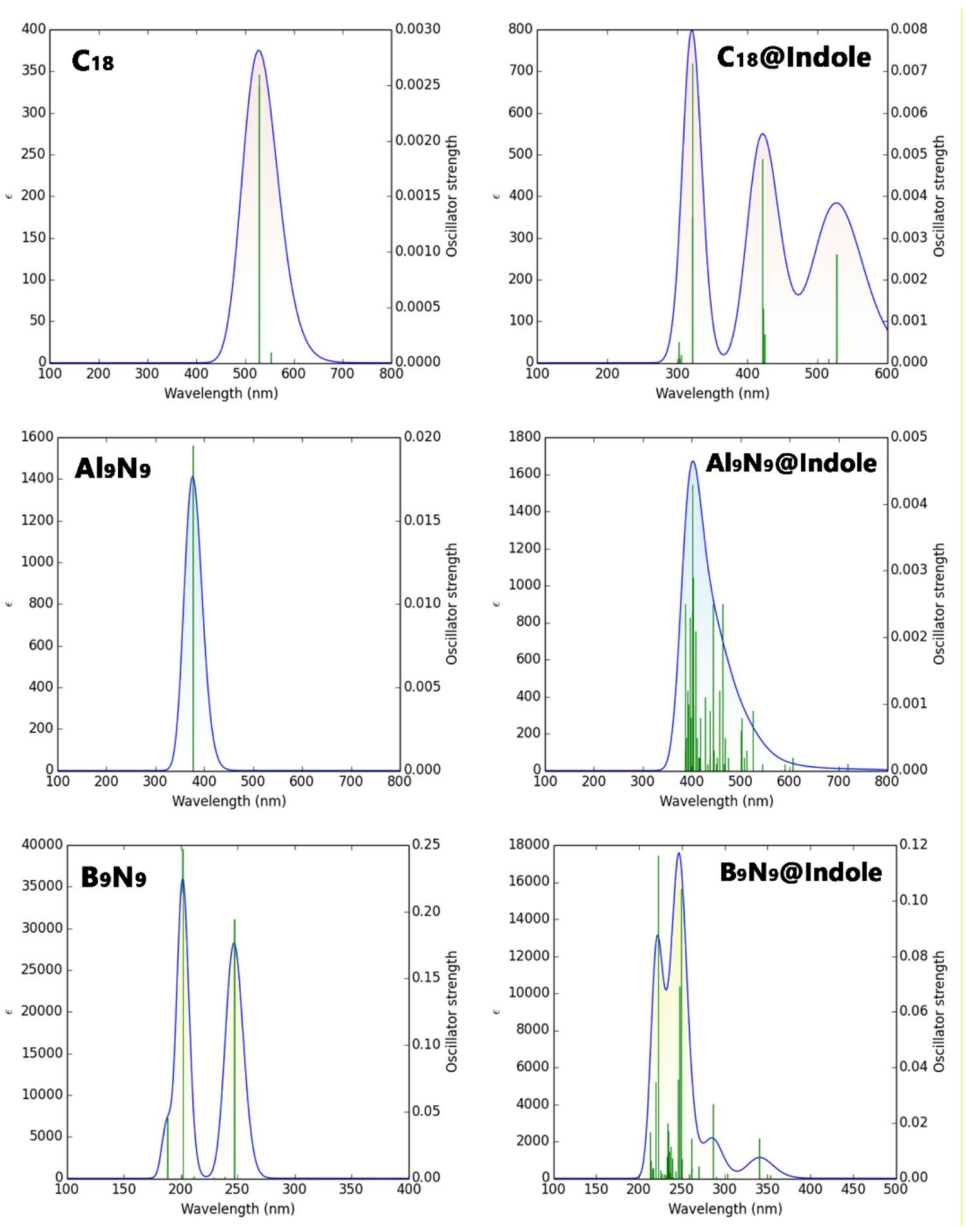
UV-spectra of C_18_, B_9_N_9_ and Al_9_N_9_ in the presence/absence of Indole.

Finally, while C_18_ exhibits a large absolute shift, it moves out of the visible range entirely, resulting in the disappearance of color. B_9_N_9_'s shift is minimal and remains in the UV. In contrast, Al_9_N_9_ is selected as the best colorimetric sensor because its significant red-shift upon indole binding (87 nm) crosses a threshold into the visible spectrum, generating a more visible and distinct colorimetric response from a nearly colorless state to a clearly colored one, which is the fundamental requirement for an effective visual sensor. For a visual comparison of the results reported in Table 4, the UV spectra of each sensor in the presence and absence of indole are shown in Fig. 9.

## Conclusion

This study examined the electrochemical and colorimetric sensing performance of C_18_, B_9_N_9_, and Al_9_N_9_ nanorings for indole detection based on the DFT, TD-DFT, and QTAIM theories. By examining various reactivity parameters (HOMO–LUMO gap (HLG), chemical hardness (η), chemical potential (μ), electrophilicity index (S)), as well as computational metrics (adsorption energy, recovery time, electrical conductivity, dipole moment, polarizability, UV–Vis spectra, and thermodynamic properties), the interaction of these nanorings with indole, a potential biomarker for diabetes, was analyzed.

The electronic properties of the nanorings in their isolated states showed that B_9_N_9_ had the biggest gap (HLG = 4.74 eV), the most stable and lowest reactivity of the three. The least stable and most reactive was C18 (HLG = 2.21 eV). Al_9_N_9_ had an intermediate HLG of 2.43 eV. Each structure exhibited a decrease in HLG in response to indole, C_18_ having the most significant change from a HLG of 2.21 eV to 1.22 eV indicating an exponential increase in reactivity. B_9_N_9_ had a moderate decrease in HLG from 4.74 to 3.41 eV and Al_9_N_9_ dropped from 2.43 to 2.23 eV.

In terms of chemical hardness, B_9_N_9_ was the hardest with a value of 2.38 eV, while C_18_ was the softest at 1.10 eV. This trend mirrored the chemical potential: C18 exhibited the highest at − 4.86 eV, indicating a strong tendency to accept electrons, while B9N9 had the lowest at − 4.09 eV.

With regard to the electrochemical sensing properties, B_9_N_9_@Indole exhibited the highest charge transfer (∆Nmax = 2.01) and its conductivity proved to be statistically significant (2.81 × 10^9^–2.88 × 10^9^ S/m) which indicates that the—significantly high electrochemical response occurred as a result of B_9_N_9_ absorbing the indole and through binding. The adsorption energy of B_9_N_9_@Indole was the highest of the three nanorings at − 20.71 kcal/mol, giving higher stability, although its recovery time exhibited relatively low stability at (1.54 × 10^3^ s). Al_9_N_9_@Indole exhibited lower adsorption energy (− 12.55 kcal/mol), and recovery time (1.6 × 10^–3^ s), and there was no significant change in conductivity from (2.94 × 10^9^ to 2.95 × 10^9^ S/m).

The UV–Vis spectra further corroborated the colorimetric analysis. C_18_ in its unmodified form displayed an absorption peak (λmax) at 527 nm, whereas B_9_N_9_ demonstrated absorption they a λmax of 201 nm, and Al_9_N_9_ displayed absorption at 376 nm. Upon binding with indole, C_18_ showed a significant blue-shifted absorption of 206 nm, from a λmax of 527–321 nm; while this is a significant blue shift, the adsorption fell outside the visible spectrum indicating that there would be a decreased possibility for a visible color change. B_9_N_9_ showed minimal blue shift of 47 nm, from a λmax of 201–248 nm, which remains in the UV, rendering B_9_N_9_ unsuitable for visible colorimetric detection. The most prominent colorimetric response was provided by Al_9_N_9_, which experienced a red-shift of 87 nm, from 376 to 463 nm, indicating a transition from the violet/UV range to the blue region of the visible spectrum. This change is drastically visible to the human eye indicating that Al_9_N_9_ would demonstrate a clear color change and would be the most applicable for colorimetric sensing.

Taking into account the comprehensive evaluation of reactivity parameters, electronic properties, and sensing performance, B_9_N_9_ would be considered the best electrochemical sensor due to its high reactivity, high charge-transfer, and detectable change in conductivity in the presence of indole. The UV–Vis spectrum of Al_9_N_9_ in the presence of indole shifts the absorption band from the UV to the visible range, making it easily detectable by the naked eye.

We hope that these theoretical findings will lay the foundation for future laboratory experiments. While our computational study provides valuable insights into the interactions between indole and various nanoring sensors, experimental validation is crucial to confirm the theoretical predictions. Future laboratory work will be essential to investigate further the practical application of these nanoring sensors in real-world scenarios, such as the non-invasive detection of indole for diabetes monitoring. The successful realization of these sensor technologies could offer new, more efficient, and accessible diagnostic tools.

## Supplementary Information

Below is the link to the electronic supplementary material.

Supplementary Material 1

## Data Availability

All data generated or analyzed during this study are included in this published article.
