# Supplementary material for: In silico study of the sensing properties of C18, B9N9, and Al9N9 nanorings for diabetes monitoring via indole detection in exhaled breath
Source: Sci Rep. 2025 Dec 24;16:3208. doi: 10.1038/s41598-025-33074-8 (PMC12830936; doi:10.1038/s41598-025-33074-8)
Supplement: Supplementary file 1 — Supplementary Material 1 [file 41598_2025_33074_MOESM1_ESM.docx]

**Structural properties**

1. **Optimized structure**

The energy profiles in the figure clearly demonstrate that the geometries used for all three systems (C18, B9N9, and Al9N9) converge smoothly toward stable minima, confirming that the optimized structures correspond to true stationary points. For both functionals (B97D/6-311G(d) and WB97XD/6-311G(d)), the energy drops significantly in the first few optimization steps and reaches a plateau afterwards, indicating that no further meaningful changes occur in the geometry beyond these steps (Fig S1). This rapid stabilization of energy (particularly for the B- and Al-doped systems) shows that the structures are not trapped in unstable configurations or saddle points but instead relax toward well-defined local minima. The agreement between the two functionals is also very strong: both exhibit nearly identical convergence trends and final energy values, further reinforcing the reliability of the obtained optimized structures. These consistent results strongly support that the reported geometries correspond to genuine minima on the potential energy surface rather than arbitrary structures.

| 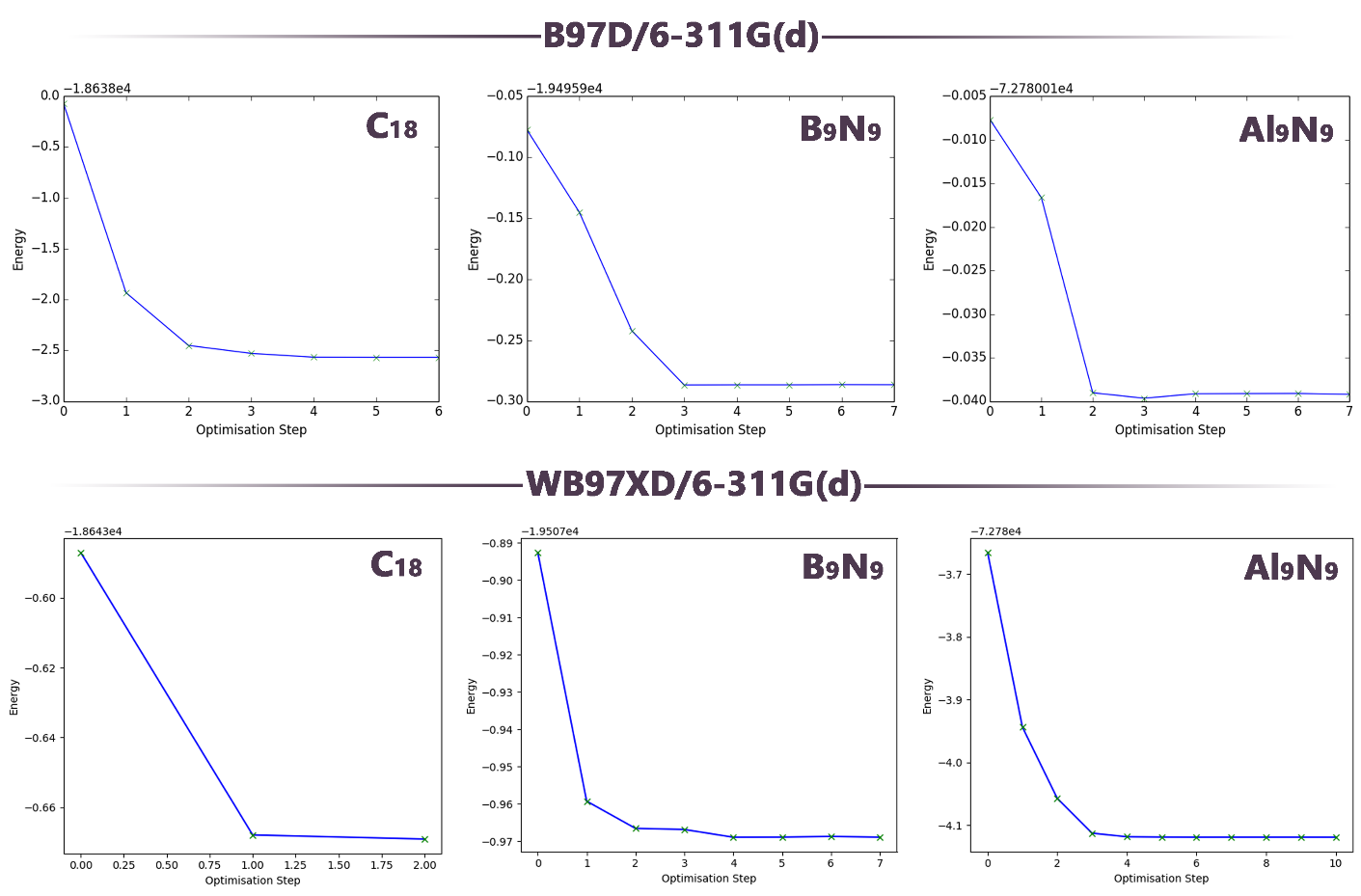 |
| --- |
| Fig S1. Energy optimization plots for C18, B9N9, and Al9N9 structures using B97D/6-311G(d) and WB97XD/6-311G(d) (showing smooth convergence towards stable minima for all systems). |

1. **Bond length/angle**

The basic geometrical features that define the properties of a molecular architecture are bond length and bond angle. Bond length ultimately is related to bond strength and sometimes to electronic characteristics such as electron delocalization, while bond angle defines overall shape and symmetry while contributing to molecular polarity. For example, deviations from the canonical bond angle may induce ring strain or minimize or maximize dipole moments, while bond length may vary in response to the quantum mechanical delocalization of electrons, as in a π-system [1]. In this way, bond length/bond angle for both C18, Al9N9, and B9N9 nanorings was modeled computationally, the results of which are presented in Table S1.

| Table S1. Calculated values ​​of bond lengths (L) and bond angles (D) between some important atoms in the designed structures. | | | | |
| --- | --- | --- | --- | --- |
| Structure | **Bond lengths (Å)** | | **Bond angles (°)** | |
| B97D/6-311G(d) | | | | |
| C18 | C=C | 1.28 | C=C | 160.42 |
| B_9_N_9_ | B-N | 1.32 | B-N-B | 138.24 |
|  |  |  | N-B-N | 178.23 |
| Al_9_N_9_ | Al-N | 1.72 | Al-N-Al | 131.14 |
|  |  |  | N-Al-N | 171.26 |
|  | **WB97XD/6-311G(d)** | | |  |
| C18 | C=C | 1.27 | C=C | 159.67 |
| B_9_N_9_ | B-N | 1.31 | B-N-B | 140.13 |
|  |  |  | N-B-N | 179.92 |
| Al_9_N_9_ | Al-N | 1.69 | Al-N-Al | 140.19 |
|  |  |  | N-Al-N | 179.92 |

The structural characterization of the nanorings was investigated using two computational methods, B97D/6-311G(d) and WB97XD/6-311G(d), and both methods provide coherent and complementary insights into the geometric features of C18, B9N9, and Al9N9. The B97D/6-311G(d) results reveal a clear progression in structural characteristics across the three systems. The C18 nanoring shows typical conjugated carbon behavior, with C-C and C≡C bond lengths of 1.29 Å and a nearly linear C–C≡C bond angle of 160°, indicating strong π-electron delocalization and sp-like hybridization. The B9N9 nanoring displays slightly longer B-N bonds (1.32 Å), reflecting stronger ionic character, while the B-N-B and N-B-N angles (138.6° and 178.5°) suggest moderate deviation from ideal planarity and slight structural strain. Upon introducing aluminum, the Al9N9 structure exhibits a substantial increase in bond length (Al-N = 1.73 Å) and larger angular distortions (Al-N-Al = 127.7° and N-Al-N = 167.8°), indicative of reduced rigidity, increased flexibility, and a potentially more reactive framework.

The results from WB97XD/6-311G(d) closely align with this trend and further support the structural interpretation. The C18 nanoring again shows a short C=C bond (1.27 Å) with a bond angle of 159.67°, confirming the presence of strain and conjugation within the carbon ring. In B9N9, the B-N bond is reported at 1.31 Å, and the angular asymmetry is preserved: the N–B–N bond angle approaches linearity at 179.92°, while the B–N–B angle remains more acute at 140.13°, indicating geometric constraints imposed by the ring size. A similar motif is observed in Al9N9, where the Al–N bond length (1.69 Å) is the largest among all structures, and the bond angles show the same pattern as B9N9, with N-Al-N being nearly linear (179.92°) and Al-N-Al remaining close to 140°. This reveals a common geometric arrangement for the doped nanorings, controlled by atomic size and ring curvature.

Importantly, a direct comparison of the values obtained from both methods demonstrates an excellent agreement. For each structure, differences in bond lengths are minimal (0.01-0.03 Å), and the deviations in bond angles are consistently below 2°, confirming that both approaches converge toward virtually identical optimized geometries. These variations lie well within acceptable computational tolerances and do not influence the structural interpretation. Therefore, the high level of overlap between the two methods not only validates the optimized structures but also strengthens confidence in their predicted physicochemical and adsorption properties. Overall, both computational techniques reveal the same systematic trend: increasing atomic size from C→B→Al leads to longer bonds, larger angular deviations, and higher structural flexibility, all of which play a key role in determining the potential functionality of these nanorings in sensing or adsorption applications.

1. **Cohesive Energy**

Cohesive energy is the energy needed to separate all atoms in a solid, essentially measuring the bond strength between atoms making up the solid material [2,3]. The study of cohesive energy is important as it is a direct measurement of structural stability and integrity. The high cohesive energy confirms the strong binding and ultimately the stability of the molecule [4]. Together, cohesive energy and vibrational analysis offer complementary insights into both the energetic and dynamic stability of molecular systems. Each of these parameters was calculated, and the results were reported in Table S2.

| Table S2. Cohesive energy values ​​and first vibration frequency for each of the nanorings studied in this work. | | |
| --- | --- | --- |
| Structure | **E_Coh_ (eV/atom)** | |
|  | **B97D/6-311G(d)** | **WB97XD/6-311G(d)** |
| C_18_ | -7.86 | -7.12 |
| B_9_N_9_ | -6.96 | -6.43 |
| Al_9_N_9_ | -4.87 | -5.11 |

The results in Table S2 provide valuable insights into the stability and vibrational behavior of the C18, B9N9, and Al9N9 nanorings. When analyzed separately for each calculation method, a clear pattern emerges. Using the B97D/6-311G(d) functional, the C18 nanoring exhibits the highest cohesive energy (-7.86 eV/atom), indicating strong bonding and high structural rigidity. This is followed by B9N9 with a slightly lower stability (-6.96 eV/atom), and finally Al9N9, which shows the lowest cohesive energy (-4.87 eV/atom), consistent with a more flexible and less tightly bound atomic framework. The WB97XD/6-311G(d) method follows the same trend: C18 has the most stable configuration with -7.12 eV/atom, B9N9 remains moderately stable at -6.43 eV/atom, and Al9N9 shows the weakest cohesive energy at -5.11 eV/atom.

When comparing the two calculation methods, both clearly predict the same order of structural stability: C18 > B9N9 > Al9N9. Although the absolute values differ slightly (with the B97D/6-311G(d) method generally estimating stronger binding energies than WB97XD/6-311G(d)) the trend remains unchanged. This agreement highlights the reliability of the results and demonstrates that both computational approaches consistently capture the same physical behavior of the nanorings. The strongest overlap is seen in the relative stability across all systems, confirming the robustness of the methodology and validating the interpretation that carbon-based rings are the most stable, followed by boron–nitrogen structures, with aluminum-doped nanorings being the least stable but potentially more reactive and suitable for functional applications such as adsorption or sensing.

1. **IR Spectrum**

Examining IR spectra is essential in molecular design because it reveals the vibrational modes and functional groups present in a molecule, confirming its structural integrity and bonding characteristics [5]. For this purpose, the IR spectrum for each of the designed nanorings was computationally studied (see Fig S2).

| 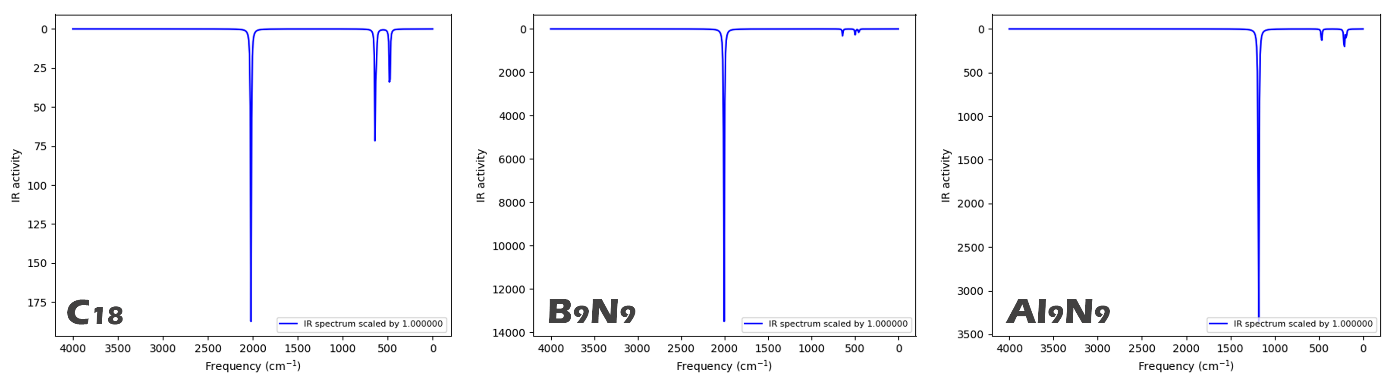 |
| --- |
| Fig S2. IR spectrum for each of the nanorings studied in this work. |

The computed IR spectra of the C18, B9N9, and Al9N9 nanorings reveal a clear progression in vibrational behaviour tied to bond strength and atomic mass. In C18, the dominating absorption peaks in the high-frequency region correspond to C-C and C≡C stretching modes, confirming strong covalent bonding and rigidity. In B9N9, the principal absorption shifts to mid-IR frequencies associated with B–N stretching and skeletal deformation, reflecting more polar bonding and reduced stiffness. In Al9N9, the strongest IR band appears at much lower wavenumber, and additional low-frequency modes (bending, torsion) emerge, consistent with weaker, more flexible Al-N bonds.

This trend is coherent with experimental and theoretical benchmarks. The strong high-frequency vibrations of cyclo[18]carbon (≈ 2000-2200 cm^-1^) have been modeled and partially observed in scanning probe and Raman studies (Kaiser et al. 2019) [6]; Hussain et al. also demonstrated tip-enhanced vibrational imaging consistent with these modes [7]. For boron nitride systems, the optical phonon in bulk h-BN lies at ~1366 cm^-1^ [8], which supports the assignment of B–N stretching in B9N9 in the mid-IR. In the case of Al-N, infrared and vibrational spectroscopy of AlN nanostructures show strong phonon modes in the 200–1000 cm^-1^ region, and nano-FTIR/TERS imaging of AlN nanocrystals demonstrates surface optical modes in that spectral window [9]. Moreover, first-principles studies of vibrational dispersion and dielectric properties in AlN provide theoretical support for the presence of IR-active phonons at lower frequencies in this material [10].

The computational results successfully reproduce the characteristic vibrational patterns expected for these nanorings. The observed agreement with available experimental and high-level theoretical data demonstrates the accuracy of the computational method employed in this work. The consistency of peak positions and intensity patterns across different systems confirms that the chosen DFT functional and basis set are capable of accurately predicting vibrational properties, validating their use for analyzing the structural stability and bonding behavior of the designed nanorings.

**Reference**

1. Shirley, W.A., Hoffmann, R. and Mastryukov, V.S., 1995. An approach to understanding bond length/bond angle relationships. *The Journal of Physical Chemistry*, *99*(12), pp.4025-4033.
2. Farid, B. and Godby, R.W., 1991. Cohesive energies of crystals. *Physical Review B*, *43*(17), p.14248.
3. Srivastava, G.P. and Weaire, D., 1987. The theory of the cohesive energies of solids. *Advances in Physics*, *36*(4), pp.463-517.
4. Hadi, H. and Shamlouei, H.R., 2021. TDDFT and AIM evaluation of the effect of H and F abstraction from the calix [8] BODIPY molecule. *Computational and Theoretical Chemistry*, *1206*, p.113494.
5. Ji, Y., Yang, X., Ji, Z., Zhu, L., Ma, N., Chen, D., Jia, X., Tang, J. and Cao, Y., 2020. DFT-calculated IR spectrum amide I, II, and III band contributions of N-methylacetamide fine components. *ACS omega*, *5*(15), pp.8572-8578.
6. Kaiser, K., Scriven, L.M., Schulz, F., Gawel, P., Gross, L. and Anderson, H.L., 2019. An sp-hybridized molecular carbon allotrope, cyclo [18] carbon. *Science*, *365*(6459), pp.1299-1301.
7. Hussain, S., Chen, H., Zhang, Z. and Zheng, H., 2020. Vibrational spectra and chemical imaging of cyclo [18] carbon by tip enhanced Raman spectroscopy. *Chemical Communications*, *56*(15), pp.2336-2339.
8. Serrano, J., Bosak, A., Arenal, R., Krisch, M., Watanabe, K., Taniguchi, T., Kanda, H., Rubio, A. and Wirtz, L., 2007. Vibrational Properties of Hexagonal Boron Nitride: Inelastic X-Ray Scattering and Ab Initio Calculations. *Physical review letters*, *98*(9), p.095503.
9. Milekhin, I., Anikin, K., Kurus, N.N., Mansurov, V.G., Malin, T.V., Zhuravlev, K.S., Milekhin, A.G., Latyshev, A.V. and Zahn, D.R., 2023. Local phonon imaging of AlN nanostructures with nanoscale spatial resolution. *Nanoscale Advances*, *5*(10), pp.2820-2830.
10. Zhang, X., Gui, W.H., Zeng, Q. and Chen, Q., 2016. Vibrational and dielectric properties of AlN: A first-principles study. *Ceramics International*, *42*(16), pp.18828-18832.
